# Patient preferences as human factors for health data recommender systems and shared decision making in orthopaedic practice

**DOI:** 10.3389/fdgth.2023.1137066

**Published:** 2023-06-20

**Authors:** Akanksha Singh, Benjamin Schooley, Sarah B. Floyd, Stephen G. Pill, John M. Brooks

**Affiliations:** ^1^ Department of Integrated Information Technology, College of Engineering and Computing, University of South Carolina, Columbia, SC, United States; ^2^ Center for Effectiveness Research in Orthopaedics, Arnold School of Public Health, University of South Carolina, Columbia, SC, United States; ^3^ Department of Electrical and Computer Engineering, Ira A. Fulton College of Engineering, Brigham Young University, Provo, UT, United States; ^4^ Department of Public Health Sciences, College of Behavioral, Social and Health Sciences, Clemson University, Clemson, SC, United States; ^5^ Orthopedic Sports Medicine, Shoulder Orthopedic Surgery, PRISMA Health, Greenville, SC, United States

**Keywords:** patient preference, shared decision making, human-factors design, health recommender system, treatment efficiency, treatment outcome preference

## Abstract

**Background:**

A core set of requirements for designing AI-based Health Recommender Systems (HRS) is a thorough understanding of human factors in a decision-making process. Patient preferences regarding treatment outcomes can be one important human factor. For orthopaedic medicine, limited communication may occur between a patient and a provider during the short duration of a clinical visit, limiting the opportunity for the patient to express treatment outcome preferences (TOP). This may occur despite patient preferences having a significant impact on achieving patient satisfaction, shared decision making and treatment success. Inclusion of patient preferences during patient intake and/or during the early phases of patient contact and information gathering can lead to better treatment recommendations.

**Aim:**

We aim to explore patient treatment outcome preferences as significant human factors in treatment decision making in orthopedics. The goal of this research is to design, build, and test an app that collects baseline TOPs across orthopaedic outcomes and reports this information to providers during a clinical visit. This data may also be used to inform the design of HRSs for orthopaedic treatment decision making.

**Methods:**

We created a mobile app to collect TOPs using a direct weighting (DW) technique. We used a mixed methods approach to pilot test the app with 23 first-time orthopaedic visit patients presenting with joint pain and/or function deficiency by presenting the app for utilization and conducting qualitative interviews and quantitative surveys post utilization.

**Results:**

The study validated five core TOP domains, with most users dividing their 100-point DW allocation across 1–3 domains. The tool received moderate to high usability scores. Thematic analysis of patient interviews provides insights into TOPs that are important to patients, how they can be communicated effectively, and incorporated into a clinical visit with meaningful patient-provider communication that leads to shared decision making.

**Conclusion:**

Patient TOPs may be important human factors to consider in determining treatment options that may be helpful for automating patient treatment recommendations. We conclude that inclusion of patient TOPs to inform the design of HRSs results in creating more robust patient treatment profiles in the EHR thus enhancing opportunities for treatment recommendations and future AI applications.

## 1. Introduction

Design of patient-centered digital health systems, specifically clinical decision support systems (CDSS), has provided a foundation for consolidating and improving clinical processes and decision making since the 1960s (1). Data-evidence based decision support systems (DSS) have been researched, developed, and applied in various clinical settings for over four decades with the application of decision trees, knowledge graphs and statistical approaches for clinical decision making (2–4). Amongst the many challenges present in current AI-based treatment DSSs is the ability to identify and include human factors, such as a range of personal preference and social determinants of health, to quantify data-evidence. As such, patient-centered and AI-powered treatment DSSs remain a work in progress. In this paper, we present the design and inclusion of one important human factor, patient treatment outcome preferences, into patient-centered clinical DSS and discuss implications for moving towards an AI-powered approach.

### 1.1. Human factors challenges in the design of health recommender systems

Recommender Systems (RSs) are a type of DSS broadly defined as information systems that are capable of analyzing previous usage behavior and making some sort of recommendations for solving new queries (5). Some real-life applications are commonly found in consumer markets such as online shopping recommendations (Amazon), music and entertainment recommendations (YouTube, Netflix) and search recommendations (Google). RSs are broadly categorized into data filtering frameworks: content-filtering, collaborative filtering, and hybrid filtering (6). For example, YouTube might recommend a video to a user based on her prior video viewing activity, or the activity of users that have similar user or viewing profiles as the index user. There are multiple variations of RSs such as context-aware systems, knowledge base systems and many applications in a wide variety of fields (6). With the emergence of AI in recent years, Health Recommender Systems (HRSs) have quickly emerged as a growing field of research (7). In a typical HRS, a recommendable item of interest is a piece of medical information such as a selected physician or treatment option. Usually, HRS suggestions are driven by individualized health data such as documented in an electronic health record (EHR) or personal health record (PHR). A subset of such HRSs is aimed at making preferred healthcare choices. The information that feed into such systems is the user profile, which could be a patient profile in a PHR or EHR in the form of a personalized health knowledge graph (8), a provider profile (9) or a combination of both (10).

One important drawback in the design of HRSs' based on artificial intelligence in the clinical setting is their lack of AI explainability (11) and AI interpretability (12) for users who are expected to make decisions based on results. Furthermore, explainable AI algorithms have been criticized for over-complicating the models to make them difficult to understand. AI techniques are often criticized for the “black box” approach (13). Among other challenges, over reliance on data represented by labels and symbols makes it harder to understand the inside working of such black box AI methodologies and systems. To create human interpretable AI systems, human factors must be included in the design such that human interactions are represented with personalized nuances of perceptions, personalities, and choices across various domains (14). HRSs for treatment support in orthopedics, for example, might refer to health data found in EHRs and PHRs including patient demographics, comorbidities, and measured mobility and function scales. This data has limited interpretability, or relevance towards making a treatment decision as it requires inclusion of human preferences, priorities, and biases that are typically used for making real-world treatment decisions. We note the case of Predict+ for predicting success of total shoulder replacement surgeries. Predict+ is a machine learning based tool created in collaboration with Exactech that is used to predict complications that result from total shoulder arthroplasty; and patient satisfaction as a result of function improvement (15). The tool used EHR data including patient demographics, diagnoses, and treatment codes. The number and types of surgical complications, and level of satisfaction, are both outcomes that are heavily influenced by factors not collected in the EHR, such as patient preferences. This and other such applications lack important patient or provider preferences as a factor in the feature set.

Recent reviews have attempted to organize the theory behind HRSs. One recent systematic literature review of personalized HRSs provides an insight into AI-methodology based classification of HRSs (16). Another discusses the applications, AI and evaluation techniques of HRSs (7) and many others discuss various aspects of a HRS including impact, target population, recommendation domain, and recommendation visualizations (17–19). However, a design science or design theory approach, providing a design framework for creating HRSs that include human factors in orthopedics remains to be seen. This is the focus of the current study.

It has been noted in recent years that a wider knowledge scope of human factors is beneficial for creating more effective recommender systems (20). A HRS inclusive of human factors in orthopedics should aim at achieving the ability to include finer nuances of provider-patient interaction, with a design framework for appreciation and anticipation of human preferences and priorities. Thus, human factors are a core requirement in the design of these systems. There are many potential human factors for a treatment decision HRS, such as patient health history, patient treatment preferences, provider biases, provider treatment profile, organizational treatment scope and constraints and finally, resources such as worker's compensation, provider availability and equipment or facility availability. The current study focuses on patient preferences regarding treatment outcomes.

### 1.2. Patient preferences for better treatment options and treatment decision making

Patients need the ability to communicate their treatment outcome preferences (TOPs) accurately and efficiently to their healthcare providers (21, 22). For this study, TOPs refer to a patient's interest in actively participating in his/her treatment decision making in a shared manner with his/her physician, particularly when multiple treatment options exist; each option having the liklihood of leading to a different set of outcomes. Different patients may prefer different sets of outcomes and thus are willing to accept tradeoffs in their treatments to achieve preferred outcomes (23–25). Currently, no existing system provides an efficient and timely approach to collect and communicate these preferences to support shared decision making (SDM) in orthopaedic practice (21, 26–28). Treatment outcome preferences may include the patient's prioritized desire for their treatment to reduce short term or long-term pain, get back to work as soon as possible, keep treatment costs low, or regain lost mobility.

Patients with new orthopaedic conditions or injuries usually have several treatment options that can affect several outcome domains (29) and patients can have different preferences over those outcome domains (21, 26–28). In addition, the orthopaedic clinical literature broadly acknowledges that treatment effects are likely heterogeneous across outcome domains across patients (30–32). Consequently, optimal treatment decisions in orthopedics are rarely “one-size fits all” and providers must help individual patients choose treatments aligned with each patient's clinical circumstances and preferences (22, 33, 34). The ability of orthopaedic patients to accurately and efficiently communicate preferences across outcome domains to their providers is vital for shared decision making (SDM) so patients can receive the treatment that best suits them (21, 27, 33). The collection and useful communication of patient preferences at the orthopaedic clinical encounter would radically transform patient-physician interaction and promote SDM and patient-centered care by allowing for patient-specific information to inform treatment decisions (35).

Despite clear patient benefits to communicating patient preferences to providers (35–37), barriers exist to capturing this communication in current orthopaedic practice workflows. Electronic medical record (EMR) systems were created for fee-for-service medicine to document the care patients received and not their outcomes and the clinical measures commonly collected in EMR systems fail to capture the range of outcome domains valued by patients with orthopaedic conditions (pain, function, quality of life, etc.) (29, 35). Thus, no existing system provides an efficient and timely approach to collect and communicate patient information on outcome domains and patient preferences over those domains to support SDM in orthopaedic practice (26, 27, 34, 38, 39). An innovative process is needed to efficiently collect orthopaedic patient preferences and rapidly communicate this information into orthopaedic practice workflows to support SDM and improve patient-centered outcomes (40, 41).

Our broader hypothesis is that using patient preference profiles as an input into an HRS will help generate more effective treatment decisions. Our prior work indicates that patient preference profiles may be an important contributor for generating patient cohort selections with greater patient similarity and assisting with patient provider communications. Patient cohort selection may also lead to improved patient understanding and more desirable treatment options for patients. This study serves as an important precursor and evidence base for analyzing our broader hypothesis.

The goals of this study are to explore patient preferences as human factors in HRSs; then design, build, and test a mobile app that collects and reports baseline patient preferences and health status across orthopaedic outcomes to the provider for use in patient care; and assess implications for HRSs in orthopaedic care. A core component of the app is a *Direct-Weighting* (DW) preference assessment approach, originated from prior research, and applied in a touchscreen based interactive design. It is envisioned that patients will use the app prior to their first visit to an orthopaedic surgeon for a new orthopaedic condition or injury. DW approaches calculate patient-specific preference weights across outcomes by asking patients to disperse portions of a hypothetical “whole” across outcomes in a manner that reflects a patient's preferences (42). DW has low respondent burden but it requires respondents to make “implicit” comparisons which may be difficult to conceptualize (42). The DW approach has become generally accepted in the quality-of-life literature and it has been shown that patients dividing up pieces of a “pie” across quality-of-life domains yields valid representations of patient preferences across the domains (42–44). However, the DW approach has not been validated with specific clinical scenarios using a clinically focused set of outcomes or by using an interactive user experience embodied in a mobile software app. Drawing on prior research, we iteratively design and develop the app with input from prior DW research, informaticians, and clinicians and test the app with patients.

The rest of the paper is organized as follows: In the methods we describe the design, development, and user evaluation of the preference app. Then we describe the thematic analysis results of the qualitative interview data, patient preference results, and results from a usability survey. In the discussion section, we connect the research objective to results and derive the need and impact of patient preferences as human factors for generating better treatment decisions.

## 2. Methods

We use a multi-method research approach to design, build, and evaluate a patient preference collection app with 23 first-time visit patients presenting with joint pain and/or function deficiency. We first identified five patient preference outcome domains that were the result of primary research by the research team. We first conceived of a list of potential patient preference outcome domains through a concept consensus building process via discussions with three orthopaedic surgeons and two physical therapists at one orthopaedic center in the Southeastern US. Three health services researchers also participated. The process resulted in the group agreeing on five preference domain areas. The research team then sought to validate the domains with patients in this pilot study. The preferences address possible outcomes, or those things that are important to a patient that she may want to communicate to her doctor regarding the impacts of orthopaedic treatment on her life. The items include asking the patient the following: “When considering treatment, it is important to me that the treatment I choose …”

• Q1. Reduces my long-term pain after treatment,
• Q2. Improves my function and ability to engage in my regular activities,
• Q3. Limits my out-of-pocket treatment costs,
• Q4. Minimizes the time required for treatment and rehabilitation,
• Q5. Limits the pain and discomfort I feel during treatment.

We incorporated these five question domains into the design of an android application to be presented to new patients in a regional orthopaedic clinic and research center. We applied a DW interaction method designed using input and feedback from orthopaedic researchers, surgeons, and experience design researchers.

We designed a mixed-method evaluation to study patient preferences using the DW approach, in which patients were asked to A. use the patient preference app, B. participate in a 30-minute interview, and C. complete a usability survey. Details of the employed methods are described below.

### 2.1. Patient preference app

**Figure 1:**
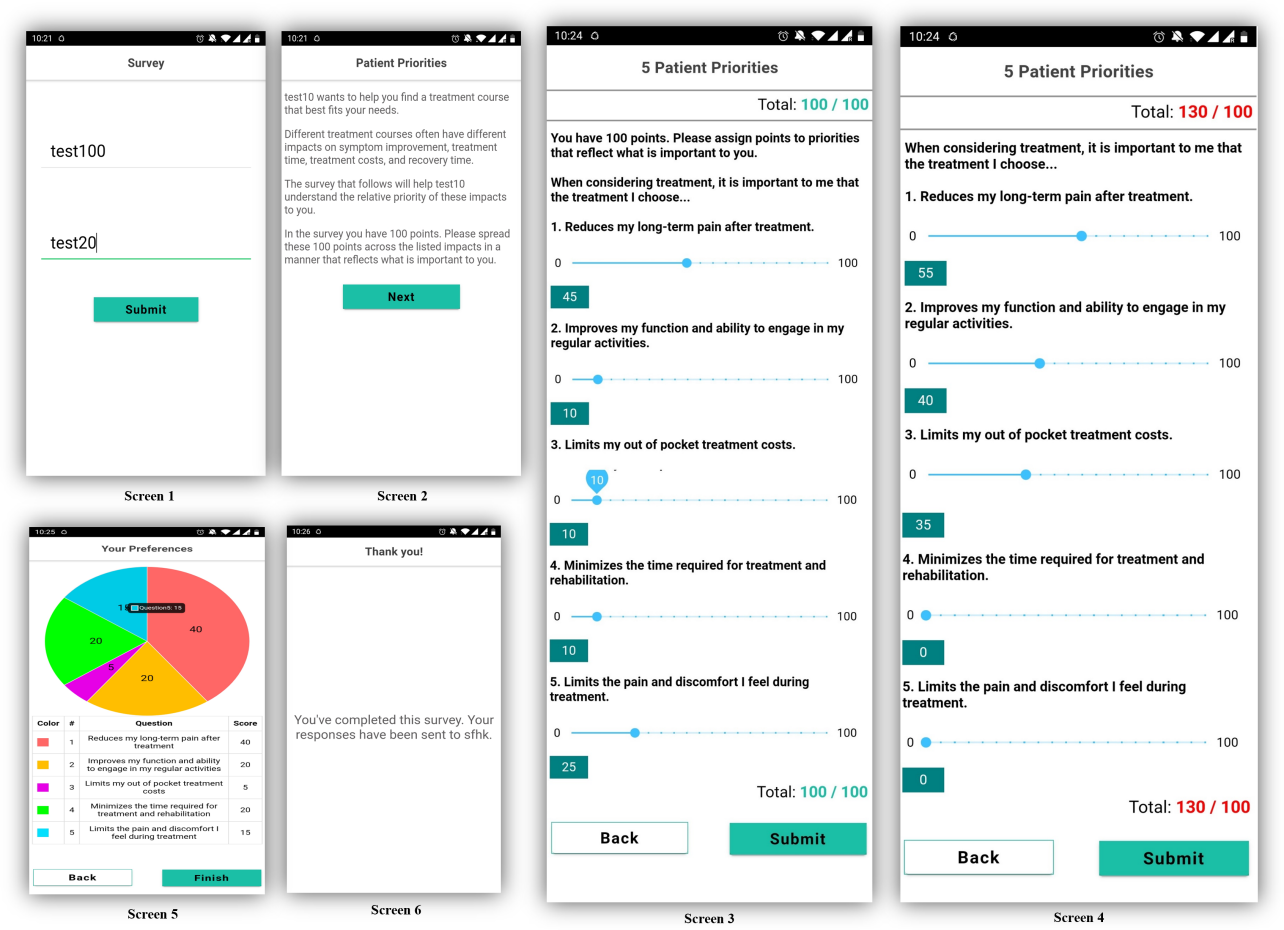
Patient preference app.

We designed a prototype of an interactive mobile application containing a patient preferences direct weighting (DW) survey and preference visualization features (see Figure 1).

Screen 1 allows the test subject to be identified as an anonymous participant of the study. Screen 2 explains the DW task to the user. Screens 3 and 4 illustrate the user's direct weighting interaction. We utilized the previously identified patient preferences and adopted a 100-point bucket weighting design in which the patients were required to distribute and assign a total of 100 points into five treatment preferences. Patients were not able to assign more or less than 100 points across domains (Screen 4). Screen 5 is a pie-chart visual confirmation of the assigned weights and screen 6 is a confirmation of preference survey completion.

### 2.2. Evaluation setting

The research setting for this study was a large orthopaedic clinic in the Southern U.S. affiliated with a large integrated medical system. We adopted a purposive, criterion sampling strategy where we contacted every new orthopedic patient at the clinic to obtain a heterogenous sample. A research coordinator contacted 100 new patients to invite them to participate in the study. Inclusion criteria included all new patients or patients visiting the clinic for new orthopaedic conditions in the age range of 18–80 years, with one or more of the following orthopedic conditions: shoulder, hip, elbow, knee, foot, hand, back and neck. Sampling occurred until qualitative data saturation was achieved. Twenty-nine (29) patients agreed to participate, with six (6) canceling prior to the interview, resulting in 23 total patients who participated in the app evaluation. Demographics of those patients who were contacted and participated in the evaluation are shown in Table 1.

**Table 1:** Participants’ demographics.

	Called	Interviewed
Total	**100**	**23**
Gender		
Female	55	17
Male	45	6
Age		
Average Age	53	57
18–30 Years	9	1
31–40 Years	15	2
41–50 Years	12	4
51–60 Years	27	3
61–70 Years	31	10
71–80 Years	6	3
80 Plus Years	0	0

Bolded numbers represent total number of patients called and total number of patients who were interviewed, respectively.

Three researchers: BS, AS and JB, all of whom are qualified health IT and health economist research experts, conducted the interviews. The evaluation was conducted 30 min prior to the regular patient check-in time of each participants' orthopaedic appointment.

### 2.3. Data collection

Data was collected in three parts. First, in an in-person setting while sitting across a table from the interviewer, each participant was handed an Android device and used the prototype mobile app to input their treatment preferences using the DW method incorporated into the app. Participants awarded a total of 100 points spread across preference outcome categories including: (1) “*Reduces my long-term pain after treatment*”, (2) “*Improves my function and ability to engage in my regular activities*”, (3) “*Limits my out of pocket treatment costs*”, (4) “*Minimize the time required for treatment and rehabilitation*” and, (5) “*Limits the pain and discomfort I feel during the treatment*”. Next, they were asked a series of questions during a qualitative interview on their perceptions of the app and the direct weighting approach. Finally, participants were asked to complete a survey containing two sections: I. A 6-item section of a custom survey instrument on app usability, patient-provider communication, patient's intention to use the app in the future, and perceptions about the treatment preference outcome domains represented in the app. The survey was designed using an implicit 4 point Likert scale where we asked users to mark between strongly agree and strongly disagree [4 = Strongly Agree, 3 = Somewhat Agree, 2 = Somewhat Disagree and 1 = Strongly Disagree]. II. A validated instrument for mobile apps, “mHealth app usability questionnaire” (MAUQ) (45) survey section containing 18-items on ease of use, usefulness and interface satisfaction. The section was based on a 7-point Likert scale (1 = Strongly Agree, 7 = Strongly Disagree). Please see Appendix A for a draft of the complete interview guide.

The semi-structured qualitative interviews touched on several aspects of patient choices and preferences as embodied in a mobile app. We asked participants questions about their perceptions of their treatment processes, as well as perceptions on the utility of the patient preference app for communicating with their provider. Example questions included:

• Please describe your general feelings about using the app.
• How do the preferences listed in the app capture the concerns that are important to you in the treatment of your condition?
• What others would you include in this list?
• What challenges do you see using this app?
• What benefits do you see using this app?
• How do you think this app (and your information that it is collecting) could be used as a part of your care?
• What suggestions do you have for improving the app?
• How has this experience affected the way you think and feel about your condition?

Questions were asked in a conversational manner to elicit deeper discussion from participants and drill down on additional topics of interest. Interviews were recorded digitally for later transcription.

All patients consented to participate prior to the study as well as in the interview. Other data collected during the interview included: date and time of interview, participant age range, gender. Interviews were audio recorded and then transcribed using pseudonyms in place of identifying information (e.g., patient name) using the format: XXN, where XX represents the interviewer code and N represents the number for each interviewer. No other patient identifiers were collected in the interviews. Each patient participant was provided with a $30 gift card as an incentive for their time and participation.

### 2.4. Data analysis

The data from in-app patient preferences was analyzed for average weights, std. deviation, maximum and minimum weight for each preference as well as maximum variation across all cases. The survey responses were analyzed for mean scores for each of the six evaluation constructs.

Qualitative thematic analysis of the interview transcripts was conducted by using a peer analysis methodology in NVivo software. For this, two researchers independently conducted an inductive analysis of data to create preliminary codebooks and reconciled these codebooks to summarize emergent themes.

**Figure 2:**
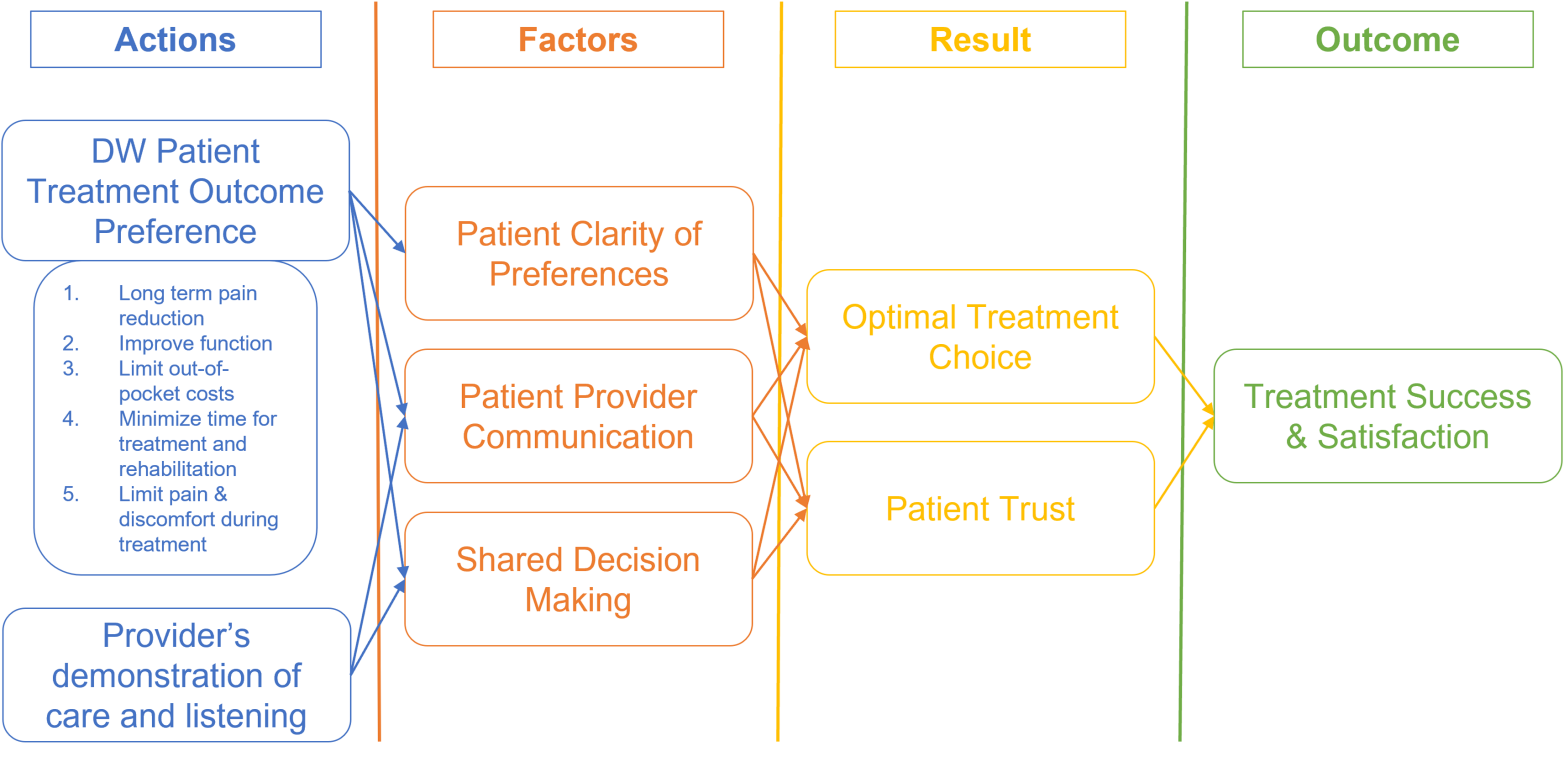
Patient treatment outcome preference—our grounded theory.

We used grounded theory (Figure 2) hypotheses to guide our analysis. The hypotheses include A. Allowing patients to express their treatment outcome preferences using a DW collection technique prior to their first visit for an orthopaedic condition induces and increases clarity of thought about the treatment outcomes they wish to achieve., B. The treatment outcome domain identified in our primary research presents the optimal set of patient preferences for their treatment outcomes., and lastly, C. Collection of patient's treatment outcomes preference improves the patient-provider communication, shared decision making and patient satisfaction on treatment decisions. We developed our interview guide based on these hypotheses and utilized this theoretical framework to guide the thematic analysis of the interview data.

## 3. Results

### 3.1. Patient preference weights

All 23 patients that participated in the study entered their personal patient preferences into the app during their in-person visit with researchers. Table 2 presents the results of their selected patient preferences as reported in the app. The most frequently weighted category was long-term mobility improvement (*M* = 33.6) followed by long term pain reduction (*M* = 28.3), limiting treatment pain/discomfort (*M* = 14.7), limiting time for treatment and rehabilitation (12.5), and limiting costs (10.8). In order to assess heterogeneity in preferences, we analyzed the extreme scoring for each question. The maximum weight that was given to each question by participants was—Q1: 45, Q2: 70, Q3: 50, Q4: 30 and Q5: 45. The minimum weight that was given to each preference was—Q1: 5, Q2: 0, Q3: 0, Q4: 0 and Q5: 0. Results demonstrate that all five core preference domains were utilized, with many users (*n* = 6) dividing their 100-point allocation across 1–3 domains. A patient attributing preference scores across all 5 preferences or setting scores close to the average was rare.

**Table 2:** Patient preference direct weighting survey results.

	Q1	Q2	Q3	Q4	Q5
Mean weights	28.47826087	32.60869565	12.17391304	12.3913	14.34783
Standard deviation	11.22444396	17.76549037	11.56268361	9.637706	9.920634
Max weight	45	70	50	30	45
Min weight	5	0	0	0	0
Patient closest to mean	30	30	15	10	15
Extreme cases	45	0	10	0	45
	5	70	10	5	10
	10	10	50	20	10
	25	25	10	30	10
	45	0	10	0	45

### 3.2. User experience and MAUQ survey results

Questions S1-S18 represent MAUQ portion in Table 3, whereas Q1-Q6 are general questions regarding the DW exercise in Preference App.

**Table 3:** User experience (Q1-Q6) and MAUQ (S1-S18) results.

Question		Mean scores	Standard deviation
Q1 (1–4)	This exercise was easy to complete.	1.15	0.387553
Q2 (1–4)	The directions were easy to understand.	1.25	0.444262
Q3 (1–4)	After reading the directions, I felt like I knew what to do.	1.3	0.470162
Q4 (1–4)	The answers to this exercise will help me to talk with my doctor about my condition.	1.35	0.587143
Q5 (1–4)	This list of concerns captured the important things to consider in selecting a treatment.	1.3	0.571241
Q6 (1–4)	I would be willing to do a similar exercise (Where I assign points to different treatment factors) for other health issues, so that I can discuss treatment choices with my doctor.	1.4	0.753937
S6 (1–7)	I like the interface of the app.	2.25	1.650359
S7 (1–7)	The information in the app was well organized, so I could easily find the information I needed.	1.947368	0.97032
S8 (1–7)	The app adequately acknowledged and provided information to let me know the progress of my action.	1.85	1.182103
S9 (1–7)	I feel comfortable using this app in social settings.	1.6	0.88258
S10 (1–7)	The amount of time involved in using this app has been fitting for me.	1.6	1.391705
S11 (1–7)	I would use this app again.	1.7	1.688974
S12 (1–7)	Overall, I am satisfied with this app.	2	1.718304
S1 (1–7)	The app was easy to use.	1.65	0.782718
S2 (1–7)	It was easy for me to learn to use the app.	1.6	1.166055
S3 (1–7)	The navigation was consistent when moving between screens.	1.85	1.083473
S4 (1–7)	The interface of the app allowed me to use all the functions (such as entering information, responding to reminders, viewing information) offered by the app.	1.6	0.810643
S5 (1–7)	Whenever I made a mistake using the app, I could recover easily and quickly.	1.842105	1.332251
S13 (1–7)	The app would be useful for my health and well-being.	1.8	1.321755
S14 (1–7)	The app improved my access to health care services.	1.85	1.423962
S15 (1–7)	The app helped me manage my health effectively.	1.95	1.460954
S16 (1–7)	This app has all the functions and capabilities I expected it to have.	1.95	1.390288
S17 (1–7)	I could use the app even when the Internet connection was poor or not available.	1.6	1.274561
S18 (1–7)	This mHealth app provided an acceptable way to receive health care services, such as accessing educational materials, tracking my own activities, and performing self-assessment.	2.1	1.735796

Results from the MAUQ survey showed overall positive results and the tool received moderate to high usability scores, patient participants agreed the most with “I feel comfortable using this app in social settings.” (*M* = 1.6, SD = 0.88), “The amount of time involved in using this app has been fitting for me.” (*M* = 1.6, SD = 1.39), “I would use this app again.”(*M* = 1.7, SD = 1.69), “The app was easy to use.” (*M* = 1.65, SD = 0.78), “It was easy for me to learn to use the app.” (*M* = 1.6, SD = 1.16), “The interface of the app allowed me to use all the functions (such as entering information, responding to reminders, viewing information) offered by the app.” (*M* = 1.6, SD = 0.81), “I could use the app even when the Internet connection was poor or not available.” (*M* = 1.6, SD = 1.27).

For general questions regarding the DW exercise in Preference App, patient participants agreed the most with “This exercise was easy to complete.” (*M* = 1.15, SD = 0.39), and “The directions were easy to understand.” (*M* = 1.25, SD = 0.44). They moderately agreed with “After reading the directions, I felt like I knew what to do.” (*M* = 1.3, SD = 0.47), “The list of concerns captured the important things to consider in selecting a treatment.” (*M* = 1.35, SD = 0.59), “The answers to this exercise will help me to talk with my doctors about my condition.” (*M* = 1.3, SD = 0.57) and “I would be willing to do a similar exercise (Where I assign points to different treatment factors) for other health issues, so that I can discuss treatment choices with my doctor.” (*M* = 1.4, SD = 0.75).

### 3.3. Thematic analysis of qualitative interviews

The usability questionnaire helped researchers understand the extent to which the preference domain questions, preference domain interactive features; and the app were usable, useful, and helpful for the participant. Interviews were conducted to assess a deeper understanding about the utility of using the patient preference app and its implications on patient care. Inductive, thematic, qualitative analysis resulted in finding several salient themes pertaining to benefits, challenges, and impacts of the patient preference app and associated impacts on the design of health recommender systems. These themes include: 1. Patient clarity in determining treatment preferences; 2. Patient preferences as human factors for informing treatment options; 3. Understanding and trust in patient provider communication and shared decision making; 4. Usability and methods to improve usability of patient preference apps. In terms of overall results, patients described a positive response to the patient preference app. Patient responses served to sustain our hypothesis that patient preference communication is important for managing patient expectations of treatment. Themes discussed below include how the app facilitated patient preference communication and played a clarifying role for understanding treatment priorities for both patients and providers alike; enhanced communication and documentation of these priorities with providers, caregivers and other stakeholders; served as a self-evaluating medium for determining patient treatment success and satisfaction based on achievement of treatment priorities during the treatment process; and facilitated trust and a positive healthcare experience. Focusing on our theoretical understanding of the orthopedic treatment process, we arranged the responses into various themes below.

#### 3.3.1. Patient clarity in determining treatment preferences

The app was reported to help patients think about how they prioritize preferences and how they would like treatment options to be personalized. Participants noted that treatment efficiency and success are related to the ability to communicate treatment expectations of the patient to the provider and the patient preference app facilitated an important precursor: patient understanding. The patient preference app was generally perceived to provide clarity in patients' minds about their preferences as well as preparing them for their meeting with the provider. One patient noted, “*This is an important meeting for the patient, and so being prepared for it means you’re going to get the most out of it, and you’re not going to do that, “Oh my God, I can’t believe I forgot to ask him that.” Which is what I often do.”* Another patient said, “*I may have thought about them [preferences], but I probably wouldn’t have said anything about them [preferences] unless the doctor actually asks you, where do you mind?”* One patient explicitly noted the intimidation felt when meeting and talking with a new provider, “*Well, it makes me think, ‘what am I going to say to him?’ Because I’ve never met him before. Am I going to be intimidated and not want to talk to him?*” This patient felt that the preference information helped overcome that intimidation. Another patient noted the need for effective communication channels between providers and new patients. “*If you don’t have that relationship with a doctor, that would be very beneficial to be able to communicate with him what your expectations are.*” said the patient continued, “*It’s [the preference app that’s] going to prepare me a little bit more, to take a little more time to think about my answer instead of… I’ve never met him. So I don’t know if he’s one of those fire off questions or if he sits and asks, listens and asks you questions. So I’m going to take more time to answer or to think about my answer before I respond.”* The use of the patient preference app helped participants think more explicitly and prepare their minds about the preference responses they would give when asked by the provider.

Some patients assume that while discussing treatment options, the provider would only focus on better orthopaedic function and not the other preferences. One noted, “*But I would suspect that along to improve my function ability, to engage in my regular activities, I’m guessing that in that consideration of treatment, that’s going to be what he’s going to bring to the table when it comes to my options for whether it’s surgery or PT or injections.*” That same patient then expressed that the app helped inform her/him about his/her additional choices of preferences.

Another set of participant responses addressed the challenges with managing patient expectations and the benefits of the app for helping to do so. One participant described how the app provided a method to begin thinking about his/her expectations.: “*It gives them a starting point,*” she said. When asked about how the app helped them think about prioritizing their expectations, one participant noted: “*Well, I mean, of course, it challenged me to prioritize and think about some things. It reinforced what my desire is, which is to improve my function and ability to engage in what I do.*” Another said, “*… it helps me feel more directive, if you will. Of directing where I want the journey to go.”* The question of what a patient wants out of their treatment was an important one for several respondents. One noted, “*… it makes me actually step back and be like okay, do I actually just want to have hardcore painful treatment and then be good for the rest of my life? Or do I want it to be a little bit more flowy? No, it definitely does spark a lot of: “Oh, what do I even want” It's really awesome.*” Another participant discussed how the app helps her self-reflect and ask clarifying questions to hone in on her treatment expectations stating, “*It kind of gives me better ideas of what to expect and what kind of treatment I will get and all, that would be the main thing.*” Another stated, “*I think that [it] really gets your brain moving and I think it gets your wheels turning and yeah, I think it's good like it is.”*

Some patients focused on better function as their major preferred outcome, “*One of the reasons I want physical therapy is I will put up with anything to try and get better function here. ….. I’ll put up with anything as long as I can get to the end.*” Participants discussed how having clarity in preference priorities helps in developing explicit expectations for their treatment to be customized to their needs. As one patient described, “*I think it’d definitely be super beneficial to show what you prioritize as opposed to… like some people might have crazy good health insurance, but I don’t. So I said limiting my out of pocket treatment cost because that's super important to me. And then being able to just share what actually matters the most, so that it can be a customized treatment for each person. So I think it's super beneficial to see each thing listed, like it's important. And then we get to decide what's most important to us.”*

Another patient emphasized the ability of the app to persuade them to evaluate tradeoffs between different available preferences, again to think about customized or personalized treatment. “*So, you made me evaluate if having pain during the treatment was more valuable than getting rid of the pain long-term and things like that,” she said. She continued, “So, making me have to deal with, again, giving the weightiness to the priorities.”* Another patient noted the tradeoffs, or the function of the app to think through tradeoffs, “*Because what it made me do is it forced me to say, “Well, the cost is not going to be as important as me getting the pain”, but still, cost is important from a standpoint of getting the value”*. Another noted, “*I’m less concerned about what the out-of-pocket treatment is. Everybody might not be in that position*”. Participants noted how the app assisted them to prioritize long-term vs. short-term pain options in a way that effectively communicates their goals. Explaining this, one patient said, “*I think it makes me… at least going through each of these makes me want to hone in on them with him [the doctor] and talk about it. How much longer do I have? You know? And what does long term look like?”*

All in all, the majority of participants commented on how the app provided a tool and method for thinking, or re-thinking priorities in a way to discuss those treatment preferences that are important to the patient. Such thinking brought personal clarity in terms of desired treatment preferences.

#### 3.3.2. Patient preferences are important human factors for informing treatment options

Participants felt that patient preferences as represented in the mobile app provided a good representation for those things that are important to them. Further, they felt that those preferences are important inputs for determining treatment options. They wanted the ability to visually correlate tradeoffs between all possible treatment options and respective patient preferences. Speaking to the importance of a patient preference acting as a valid data point, one patient said, “*Okay, this is my option based on the data, this is the best option. And the doctor is not just basing it on their own experience, but on the experience of the collective data. Yeah. Statistics.”* Similarly, one participant discussed the importance of being able to retrieve his preference profile data from the app database at any time to review and reassess, “*it's very beneficial for me… ..To be able to access all my information like that.”*

Participants discussed their appreciation for the ability to set, view, change and communicate preferences across time. One said, “*to be able to look back and see what your preferences were at different points in time with the doctor could also be of value.”* Referring to the same functionality, another patient affirmed wanting to review and possibly change preferences in the future. She said, “*Yes [I would review or change my preferences in the future], but I think it would be after, after my first visit, because sometimes you go and you think, okay, they gave me all this information, but I didn’t ask if am I going to have a lot of stitches? Am I going to … I’m allergic to nickel, so they can’t use staples. They have to use glue or they have to use sutures. Those things would probably come after I would have these …I would have questions after I had time to process some of the information I had.”*

Patients noted that treatment efficiency can be achieved by better understanding the goals of the treatment and how those goals can be met during the treatment. One participant stated, “*I like to understand why and what they’re [the doctors] going to do. That's really important to me. When I’m finished with this process, I want to know, or when I see my doctor, I want to know what his plan is? What should I expect from that plan? Yeah. That's about it. And what's he going to do?*” In this regard, patients related how keeping track of treatment options and choices can help patient understanding and treatment efficiency.

Many patients appreciated the completeness of the preference choices domain in the app with positive statements. Participants found the list of five outcome domains adequate, sufficient and complete to convey their priorities associated with choosing a treatment. For example, one participant said, “*Function, pain, cost, time and discomfort. Yeah, those are the big ones that hit me off the top of my head*.” The list of outcome domains were said to have been specific enough to be distinctly different from one another and well understood. In addition, the use of the app survey instrument helped patients to get understanding and clarity about their priorities associated with treatment choice. As noted by another participant, “*… These [categories] actually, when I first looked at it, I was like, oh God, I’ve got to rate these, and they’re going to be so close and similar that it's going to be hard to rate them. But actually, these were very specific.*” Overall, participants found the list of outcome domains to be complete. Some participants also expressed the need to have a discussion with their doctor about the treatment tradeoffs that would help them accomplish the best mix between their multiple preferences, and also, mechanisms to measure treatment success against the preference indications made in the app.

When asked about suggestions to add to the list of preferences, few patients noted the less frequent provider visits and less average number of provider visits required for the treatment as their preference. “*I can’t think of anything, no. Maybe even limit my actual doctor visits, but that kind of ties into that. Or limits the time in between my visits, because this is months and months that I had to wait in between each visit with my PA and my surgeon, was seven weeks later. So I would say the quickness of my treatment would be a good one. Like the speed through which I finish it all.*”

One patient noted the need for the ability to indicate a preference for maximum value treatment, if they are provided similar treatment choices. “*If I had to think of one thing, I would say the biggest bang for the buck. So what treatment is going to maximize in the minimum?*” They continued, “*Time and cost. That's going to allow me to re-engage at my normal level of activity. What's going to give me the max for the minimum? So what is the max I need to put in? Do I need to go big upfront to get the max? Or is it going to be incremental?*” The patient concluded by summarizing their suggestion as finding the answer to the question: “*Well, what's going to be that Delta? What's going to be that sweet spot?”*

#### 3.3.3. Understanding and trust in patient provider communication and shared decision making

The patient preference app in general enhanced patient's trust in the treatment, in the provider, and with the clinic. One participant said, “*I think it enhances my view of the practice in general.*” Several other participants noted similar sentiment that the preference app provided a reassurance to them that the providers care about their concerns and want to understand their treatment priorities. This increased feeling of trust led patients to feel more confident in the treatment choices, shared decision making during the patient visit, and eventually treatment success and satisfaction.

Patients also noted that shared goal setting for the treatment increases with communication of patient preferences. “*He should be able to say, “It's going to be three months before you…” I had a knee replacement. I said to my doctor, “When is the pain going to stop?” He said three months. It was to the day practically when he told me. I could do it. I just wanted to know when it was going to be over. That was the kind of stuff I wanted to know.*” Another patient notes, “*Probably at that point, I want to know what he's going to do and what the outcomes should be. Farther along in the process I want to know about pain, I guess. But I think, I guess I can say when I had my knee replacement, he told me that we’re going to do a nerve block and we’re going to do this and this and this. And we’ve tried giving you… I mean, they’ve done some research and we want to try giving you Tylenol and big doses while you’re here. Okay. And if that doesn’t work, there's some other options. I guess just everything about the process. How many days will you be in the hospital? How many days will you not be in the hospital? How many times will I see you when I see you back.”*

The preference app was noted in aiding to bring patient and provider on the same page by bringing clarity in exchange of patient preferences and expectations with provider understanding of problem and treatment options. “*I think it would be beneficial, again, like I said, to ensure the patient and the doctor are on the same plane as far as, if you’re having a surgery, your pre-care and your after-care. Make sure you’re on the same wavelength as far as what you'e expecting.”* Another patient noted, “*Somebody calls you up and asks you, so I think you can say, ‘Hey, I’ve got this great app that you can use to make sure that you and the doctor are on the same wavelength.’*”

Another key benefit noted by patients in terms of patient provider communication and shared decision making is the evaluation of efficiency based on the shared goal setting after communication of patient preferences. Patients noted that preference app helps in enabling them to evaluate how their personal treatment goals were communicated, understood and implemented into treatment, as well as the overall efficiency of chosen treatment as a measure of “treatment success” and “treatment satisfaction”. A patient spoke about evaluating the treatment after the point of preference communication “*… from the standpoint of rating the doctor at the end. How did he do?”* Another patient noted, “*It's all written, but it's a good way to, again, grade your therapist, your therapy*” One patient stated the ability to explicitly evaluate the provider and treatment in MyCharts and noted that preference app will add to streamlining the treatment evaluation, “*Yes. I mean, I use it in, I put most of my stuff in MyChart. They send surveys and, I don’t know whether it’s MyChart related, but [the health system] sends a survey after every doctor visit. How did the doctor do, how are the nurses, receptionist, et cetera. And I think that’s a good tool for any organization to gauge how their people are doing and also to come up with means and mechanisms to do better*.”

Participants noted the clarity and the enhancement the use of a preference app brings to their communication with their provider. Participants noted ease in communicating difficult topics such as money constraints, as a participant noted, “*… then also, if it’s in the app, then it’s in the patient’s mind too, to discuss even if the doc doesn’t bring it up …*” Participants also noted that the app brings about their most important concerns to the table such that the discussion with the provider during visit revolves around that concern, thus bringing more focused treatment options specific to their needs. A participant quoted “*… and it eliminates the fear for them so that they can communicate. And then hopefully when they get there, the doctor is able to ease their stress and say, “Oh, okay, let's look at your long-term pain treatment or so-and-so and so-and-so,” and they’ll be like, “Oh my. You're reading my mind. I feel so good about this. This appointment …” because a lot of people don’t. They don’t think their doctors care*.” Another said, “*And so if it's available to the doctor before you get to the visit, they already know what you need*.” Participants also agreed that communicating their treatment preferences with their doctor is improved with the use of the app, saved time during the visit and helped them focus their communication with the providers.

Participants discussed the transient nature of patient-provider relations, relating the importance of having a record of communications to share with providers as patients move from one to another. One stated, “*Because you know, you see somebody else, this guy retires and you see the next guy. Oh, what did they do? Well, they did this and they did that. And then they said that that was what they needed to do at that time. I don’t know*.”

#### 3.3.4. Usability and methods to improve usability of patient preference apps

The patient preference app was described by participants as easy to use and simple in its overall functionality. For example, when asked about difficulty using the app, one user stated, “*No. Pretty easy. I mean, I’ve had two strokes this year, and I had no problem navigating it …*.” One patient noted the clarity brought on by the preference visualization at the end of the app survey explaining, “*I liked the visualizations. I liked it because of the color. So, the color made it clear for me. It let me see physically what my choices are.”*

Several patients likewise noted how the chart formatted visualization helped them to “clearly reaffirm responses before sending” to their providers. Patients also noted positively the ability to modify their responses at the final step before submission, which could be accomplished as many times as needed until visually satisfied with their direct weighting assignments.

Patients also discussed ways that the app could provide a more usable experience. Some patients noted the need to link it to EHR software utilized during the scheduling of the appointment and subsequent treatment visit. For example, one patient noted, “*I think the app itself was fairly simple and self-explanatory. Just I think the capability of it linking with MyChart [patient portal] or being able to cross link with other MyCharts would be very beneficial.”* While usability was generally high, ease of use was reported to reduce with increased age.

The amount of time required to complete the preference app survey was pointed to as a valuable signifier for improving the usability of the app. There were mixed responses in this regard with some believing the process was efficient while others thought improvement could be made. As one participant noted, “*I mean, that part of the thing, if there's more to the app than just that, then that's fine. I mean, it takes two or three minutes*.” Most people were able to complete the app survey in 2–5 min, however, there were exceptions as well where participants struggled with calculating the total weights on the fly.

In this regard, several patients discussed the usability of the direct weighting mechanism used in the app. One noted that some instructions might have been beneficial for understanding how to complete the app survey. Another patient expressed difficulty in following the instructions and suggested a more engaging strategy by dividing the instructions into multiple screens, so that the information on each screen is reduced.

A few patients noted the familiarity of using a Likert style scale over the DW technique used in the app, one noting, “*Most of the surveys I’ve ever taken, you answered them with a one, 1 to 5 or 1 to 10 in response in terms of how important they were.”* A different patient described how the expression of emotion as potentially more important than numerically assigning weights to preferences. The patient stated, “*As them even being able to…maybe rating and ranking it from red being pain, to green, being not as much pain. Being able to use colors, to tell the intensity of the pain. Be able to express what's going on, what they’re feeling. Yes. Versus it just being assigned numerically for them.*”

Participants discussed challenges and benefits of the interactive DW approach. In general, patients discussed that the use of the 100-point constraint in the DW mechanism felt unfamiliar for a survey-based instrument, though the DW scoring also served its purpose to help patients compare and contrast different preference types and bring clarity to their thoughts about their preferences. One patient discussed her thought process while trying to figure out how to distribute points across categories, “*… because I read all five [preference domains] first, and then I went and said, okay, if I had to rate this, I’m going to put this as 60, because this is the most important to me. But then I knew that I was going to have to start altering that what was most important to me down and use my points to then kind of discern what was my least important and what was my most important, and then determine how that fit into the whole graph.*” This patient's summary sentiment was that his thought process assigning direct weights made for a more accurate and personalized score. Other patients noted that the self-evaluation inherent in the app was helpful, with one explaining, “*So you made me evaluate if having pain during the treatment was more valuable than getting rid of the pain long-term and things like that. So making me have to deal with, again, giving the weightiness to the priorities.*” Another patient thought the use of the app helped her bring stark clarity in her preferences and noted no need to modify the design of the app as it was very clear to her noting, “*I think that really gets your brain moving and I think it gets your wheels turning and yeah, I think it's good like it is.*”

In general, patients found the DW mechanism to be thought provoking yet also required more effort than expected while applying math “on the fly.” Some older age participants found the DW approach more difficult in terms of allocating and totalling100 points across 5 domains, as well as feeling less familiarity with the technology. Overall, a few patients noted the need to reduce the DW complexity in the app.

One patient stated, “*I think the numbers … I got confused counting it all up. Does this fill in and then you fill in the rest or is it each one is its own?*” Another patient noted, “*If you’re considering an older person, I’m older, an older person, then you don’t want to have the person having to do the additions.*” On the other hand, a younger patient expressed the need for a higher total score so that she could be more specific in her assignment of weights to preferences stating, “*I didn’t find any challenges using the app, no. I would have liked to have more bandwidth, have more bandwidth for the communication. All I had was 100, so I may have wanted to have maybe double that or something like that so I could have been more specific with my responses*.”

Suggestions for DW interface interaction improvement included instantiation of a token/points oriented DW preference scoring methodology where numbers could be directly input from the device number pad, rather than a 1–100 sliding scale approach. Participants noted that such a change could help improve preference weighting cognition and shared decision making with the provider.

On being asked about a possible solution, patients noted the need to modify the interface according to user's age groups. One suggested, “*So depending on the age group that you’re working with, you might have to make some adjustments If you’re working with this app, let's see, you had a way to say pick the age group and modify it according to the age group, 18 to 20, 40, 50, whatever and then you’ve made the modification and the 18 to 20 year olds shouldn’t be checking in that area where it's 50 to 100 or 50 to 80 years, or whatever. You can make that modification.*” Another patient suggested modifying the highest possible score with the DW technique according to the age group, “*I think you need to reduce the numbers for the older [patients] and tell them to write it in if it's more than 50 or more than 25, write it in.*” And,, “*… *[lowering the total number from 100] *would help them instead of them seeing that number in a hundred and getting that number stuck in their head, because you could say 25 is the highest you can go.*”

Suggestions for interface improvement were also discussed to enhance usability and comprehension of the instructions on screen as some patients found some difficulty following the instructions provided on the app screen. One struggled with understanding the total weight limit noting, “*It wasn’t very clear that I couldn’t go over a 100.*” Another suggested simplifying the instructions and the wording of the preferences stating, “*I don’t know how to change it, but I feel like … if you worded it … a little less wordy, if that makes sense.*” One patient explained that they preferred prioritization of preferences over assigning weights to preferences, “*I think the prioritization is a better method*.” A few patients also noted assignment of coins or smiley faces or other such icons representing points across preferences might make the DW technique easier. One patient noted he would prefer filling in the weights directly in a text field instead of having to slide over within a specified limit. Some patients expressed the need to be able to modify responses at a later time in case they changed their minds about their preferences, with one responding, “*Just because I know I have to fill some stuff like this out for my physical therapy and sometimes I don’t think that way anymore and I wish I could go back, but I mean I can communicate that to them, but I wish I could go back and change it so that it looks different.*” Likewise, another patient noted, “*I would say only thing added is like the ability to change your answer. If you fill it out a week before, and then you were just in a ton of pain and you’re like, you know what, I do want to limit my pain, to be able to go back and change it*.” Furthermore, when asked about what other function patients might like to have in the app, some patients described the need to communicate with the provider through the app in preparation for their visit, with one explaining her reasoning, “*Well, if I need to take like a pain med before I come or certain things I need to put on like leg braces or arm braces or not put them all on things like that*.” These and other suggestions described less frequently by patients were recorded and prioritized for future consideration.

## 4. Discussion

There are several treatment outcome tradeoffs that could be made during the process of an orthopaedic treatment, and these have been presented in this paper. These options include reducing long-term pain after treatment, improving function and ability to engage in regular activities, limiting out of pocket treatment costs, minimizing the time required for treatment and rehabilitation, and limiting the pain and discomfort I feel during treatment. This app presented in this research has sought to bring some clarity to the patient in understanding these options through the design, development and testing process. Many patients want personalized treatment, vs. a standardized treatment that may or may not fit their needs, that considers personal health history and experiences with different providers and treatment outcome options. As a result, patients may want their healthcare providers to communicate and enquire about those priorities during their visit. Providers also understand the importance of discussing priorities with their patients as being related closely with patient satisfaction. Due to limitations of time, lack of familiarity with a new provider, the pain and the discomfort caused by the patient's orthopaedic condition and other such factors, patients may not prompt that opportunity. However, their expectation for a *successful* treatment innately includes their priorities. This may create a mismatch in the patient's priority of preferences vs. the priority of preferences communicated to the provider. Participants in this study validated these concerns and issues and provided evidence as to the potential benefit of digital health means to help bridge a communications gap. Based on the study findings, the paragraphs below provide a discussion into five key areas that extrapolate on these concepts concerning our hypothesis that patient preferences are an important human factor for determining treatment options and suggests further the importance of facilitating patient-provider communications and shared treatment decision making.

### 4.1. Primary findings

The average mean scores for all survey questions leaned heavily towards *Agree* or *Somewhat Agree* indicating positive perspectives towards the app in terms of usability, acceptance, patient-provider communication, and completeness of treatment preference outcome domains. For the survey results, the highest scores (strongly agree) were selected for the question on the ease of use of the app procedure validating the simple and efficient design of the app from the patient's perspective, indicating a positive acceptance of the app design. Lower scores were given to the question on interest in using a similar app for other healthcare conditions, indicating some agreement that the utility of the DW interaction is valuable enough to use more broadly, including the need to integrate the DW preference app into the EHRs. Qualitative interview analysis confirmed these results and are further discussed below.

#### 4.1.1. Clarity in determining patient preferences

Among the many benefits of inciting a discussion about patient treatment outcome preferences, this study serves to validate the notion that the patient preference app as currently designed may help promote clarity about patient outcome preferences for patients. Based on the thematic analysis and results from interaction with patients, we note that the app demonstrated usefulness in causing patients to think about how they prioritize their treatment preferences and how they would like treatment options to be personalized. This thought process may further help patients to manage their expectations regarding their treatment. This may be especially important in a world where an increasing number of patients have high expectations regarding their treatment and recovery (46–49). For the participants of this study, the app seemed to help patients understand treatment outcome tradeoffs and how their preferences in this regard affect their treatment choices. While treatment and outcome tradeoffs may exist in healthcare (50), we also found that there are trade-offs to be made in the design of the direct weighting (DW) technique in the app to help provide clarity in the patient's mind regarding their treatment options, including perceptions about their preferences and potential outcomes. Benefits of DW include persuading the patient to think about their expectations and really understand their chosen treatment preference domain. The app also may assist in bringing clarity to patient provider discussion during a patient visit, though this has yet to be tested In the limited amount of time spent during the patient visit, the clarity achieved regarding patient preferences may result in creating a shared understanding between patient and provider. The patient preference app may also help with a patient's goal setting for her own treatment and recovery and thus ultimately increase patient confidence in treatment decision making.

#### 4.1.2. Patient preferences are important human factors for informing treatment options

Human factors reference human emotions, behaviors, and cognitions related to the design, adoption, usage, and implementation of health technologies (51). Through this study we posit the need to include patient preferences as human factors for informing treatment options and create processes and technologies that facilitate this notion. Patient preferences provide invaluable social determinants of healthcare as individual preferences reflect personal sentiment and goal making—powerful constructs for determining positive health outcomes (46, 52, 53). Technologies that can accurately collect, communicate, and analyze patient preferences provide an important contribution to the informatics literature (54).

This study helped validate the completeness of outcome preference domains for orthopaedic treatment. Findings may also be useful for extending the use of these preference domains into other health specialties, perhaps with the most logical extension being other types of surgeries. In terms of the orthopaedic patient outcome preferences captured in the DW app, the mix of weights assigned by each patient differed across all patients indicating that a high degree of preference variation exists across patients. We concluded that participants demonstrated having a distinct combination of treatment priorities that was adequately captured across the five domain options. Pain alleviation, for both during the treatment and in the long-term, was the most heavily weighted preference across all patients. However, cost of treatment and time taken during treatment were also found to be important to many patients. The participants in this study demonstrated concern about their personalized needs as indicated by the heterogeneity of DW responses across preferences, suggesting the need for a tool such as this to capture and communicate such specificity to physicians. The patients also indicated a need to connect their preferences with treatment options, which further validates the need for collecting and analyzing preferences to facilitate relative decision making.

#### 4.1.3. Patient provider communication for shared decision making and personalized treatment options

Findings from this study indicated that the preference app may help develop patients' trust in the healthcare facility, in the quality of service provided, as well as in the provider's understanding of their condition and treatment expectations. These are particularly useful findings for a few reasons. First, common healthcare quality measures include patient satisfaction. Increased patient trust may translate into more satisfied patients, an important goal for healthcare organizations (55, 56). Second, treatment goal setting is an important function during a patient visit and study findings indicate that the preference app may facilitate this process by conveying the preferences of the patient and contributing to shared decision-making. The enhanced patient clarity about personal preferences could be communicated in advance to the provider—at least that is the intention of this app.

This process, from the perspective of patients, could serve to enable communication during the patient visit to be directed towards setting treatment goals and plans, rather than spending valuable time discussing patient preferences. Indeed, participants noted the potential for the preference app to bring the patient and the provider to a common understanding about the patient's needs, enabling shared decision making.

#### 4.1.4. Usability and methods to improve usability of patient preference apps

This study evaluated aspects of usability for the preference app as well as identified methods to enhance the interface to improve usability for various age groups and varied user requirements. One notable suggestion from participants was to improve the app instructions by breaking them into multiple pages, or to introduce an audio component to talk through the instructions to achieve a reduction in the instruction per page ratio. Patients also noted the need to enhance the app to consider the time needed to complete the preference survey. While these suggestions refer to the time and ability of the user to understand their desired preferences and assign representative weights, it also induces a thought process in the patient's mind regarding their preferences for treatment. Some patients expressed a need to go back and change their preferences as they were not satisfied with their responses made in the first attempt. This need further elongates the time taken to usVe the app and to indicate preferences. We consider this *time efficiency* vs. *user contemplation* as a valuable user experience design tradeoff allowing patients to arrive at a point of intellectual clarity regarding their preferences. Patients in this study who were already predetermined about their treatment preferences expressed an ability to finish using the app quicker than patients who needed time to think about their preferences, which further establishes the ability of the app to help bring clarity to patients about their outcome preferences.

Another tradeoff to consider in the user experience design is balancing clarity in patient preferences with the potential difficulty in assigning weights with the DW mechanism. While many patients found it easy to specify the weights to the preference domains, some indicated a preference towards different methods, such as stack prioritizing the five preference boxes, directly inputting numbers, or assigning weights in the form of coins or other relatable icon (e.g., smiley faces) instead of numbers. Some patients found the numbers to be an intuitive method to assign weights while others indicated that numbers made them engage more and do more mental work to assign correct weights to their perceived preferences. While prioritization may be another useful way to express the priorities in preferences, we note that it is less specific than the DW technique utilized in the app, as it does not allow for two preferences to have the same priority. Many of these patients noted that the visualization at the end of the survey helped to evaluate their choices and understand their preferences. The ability to modify preferences was also appreciated by several patients.

Some reported difficulty adding up the domain totals when using the DW app survey as a result of age, or due to a lack of familiarity with the technique/technology. We concluded that modifying the DW score complexity might enhance the ease of use for older age groups. Integration of patient suggestions for alternative interface components may help to improve the user experience in future versions. Largely, patients found the app with DW technique to provide a simple and beneficial method for communicating patient preferences to their doctors, for building trust in the treatment process, and to participate in shared decision making with their providers. Patients validated the ease of use, sufficiency and completeness of the treatment preference outcome domains, highlighting that the preference app captures the most important patient priorities through the DW technique.

### 4.2. Implications for health recommender systems

The patient preference app provides a basic foundation for having the ability to set, view, change and communicate preferences across time and space for both patients and providers. When considering the design of recommender systems for healthcare (HRSs) (17), there is a need to correlate tradeoffs between options while also considering all possible treatment options. Participants in this study demonstrated that patient preferences are important for determining the most relevant and effective treatment options. Integration of these preferences into the design of HRSs may address the patient's personal needs and preferences in a more efficient manner that reduces patient-provider discussion time while also honing in on the most important factors for patients. Human factors built into HRS visualizations may also provide a means for presenting predictions on which data to present in the future to patients and providers for treatment options. Integrating patient preferences into the design of HRSs and treatment options may help influence the treatment choices and decisions which in turn brings patient understanding and satisfaction in these treatment decisions. This may further help in promoting treatment efficiency across time as well as accountability for the treatment goals set within the shared space of patients and providers.

We found the preference app also provides a potential framework for treatment efficiency evaluation. While this needs to be further explored in future studies, we believe that the patient preference outcome domains represented in the app provide key measures to evaluate fulfillment of patient treatment goals that associate with patient preferences. Inasmuch as patients and providers want to evaluate how treatment goals are communicated, understood, and implemented, as well as the overall effectiveness of a chosen treatment as a measure of “treatment success” and “treatment satisfaction”, the preference app data may provide a framework for such evaluation. An HRS would require such an evaluation framework in order to provide information and predictive value to users. These measures may also relate closely to the value and importance of shared decision making and the ability to evaluate such. The app in this study provides a framework for the patient to visualize their preferences over time and correlate those preferences with the progress of their treatments over time, thus providing an evaluation of treatment outcomes while managing treatment expectations.

### 4.3. Limitations and future research directions

This study is limited to one orthopaedic practice location in the state of South Carolina. The study was limited to 23 patients at this practice, making the purposeful convenience sampling somewhat limited due to the location constraint and the patients who on their own accord made appointments with the practice during the study period. Although our patient recruiter made significant efforts to achieve a representative sample, 23 patients overall cannot represent the entire population of new orthopaedic patients. Nonetheless, the sampling was effective for an early phase translational design, feasibility, and user study. A larger number of patients may provide more varied demographics and broader insights. We also suggest a larger study in the future for a more inclusive analysis of patients across different locations.

Furthermore, a broader study is also needed to study the correlations between the preference app as a social determinant of health, including human factors for treatment and treatment “*success*” measures such as patient satisfaction, patient understanding, shared decision making and treatment efficiency. The impact of designing preferences as human factors in HRSs for treatment options also needs to be studied further.

## 5. Conclusion

We conclude that patients found the DW patient preference application in this study to provide a simple and beneficial tool for communicating patient preferences with their providers, for building trust in their treatment and for participating in shared decision making with their providers. Patients validated the sufficiency and completeness of the five treatment preference outcome domains, highlighting that the preference app captures the most important priorities adequately as well as defining human factors for the design of patient-centered decision support systems. Further, the patient preference domains, associated data collection, decision support capabilities, and communication and decision making value offered to patients and providers provides a foundation for designing AI oriented health recommender systems in the future.

As patient preferences become more integrated into the care process for patients across a broad spectrum of health conditions, these results provide evidence for a DW approach and interactive design for patients to communicate their treatment preferences to their providers, and further need for evaluation of this approach across healthcare domains and regions as a valuable component of patient-centered engagement and quality care.

## Data Availability

Unfortunately, post publication, approved IRB protocol dictates that we destroy the interview files. However, survey data can be made available upon request to the corresponding author/s.
